# Effect of endosulfan on immunological competence of layer birds

**DOI:** 10.14202/vetworld.2016.777-782

**Published:** 2016-07-28

**Authors:** P. P. Singh, Ashok Kumar, R. S. Chauhan, P. K. Pankaj

**Affiliations:** 1 Animal Science Section, Krishi Vigyan Kendra, Rajmata Vijayaraje Scindia Krishi Vishwavidyalaya, Morena, Madhya Pradesh, India; 2 Department of Livestock Production and Management, College of Veterinary & Animal Sciences, Govind Ballabh Pant University of Agriculture and Technology, Pantnagar, Uttarakhand, India; 3 Section of Transfer of Technology, Livestock Production Management, ICAR-Central Research Institute for Dryland Agriculture, Hyderabad, Telangana, India

**Keywords:** blood biochemistry, enzyme-linked immunosorbent assay, layers, immune competence, organochloride, teratogeny

## Abstract

**Aim:**

The present study was aimed to investigate the immunological competence of endosulfan insecticide after limited oral administration in White Leghorn layer chickens.

**Materials and Methods:**

A total of 20 White Leghorn birds were given endosulfan in drinking water at 30 ppm/bird/day (no observable effect level dose) for a period of 3-months. Immune competence status of layer birds and chicks hatched from endosulfan offered birds were estimated at 15-day interval in layer birds and at monthly interval in chicks using immunological, biochemical parameters, and teratological estimates.

**Results:**

There was a significant decrease in levels of total leukocytes count, absolute lymphocyte count, absolute heterophil count, total serum protein, serum albumin, serum globulin, and serum gamma globulin in the birds fed with endosulfan as compared to control. Similarly, immune competence tests such as lymphocyte stimulation test, oxidative burst assay, and enzyme-linked immunosorbent assay tests indicated lower immunity in birds treated with endosulfan as compared to control. Subsequently, chicks produced from endosulfan-treated birds were also examined for immune competence, but no significant difference was observed between chicks of both the groups.

**Conclusion:**

The exposure to endosulfan in limited oral dosage was able to exhibit hemo-biochemical and other changes that could be correlated with changes in the immunological profile of layer chickens suggesting cautious usage of endosulfan insecticide in poultry sheds.

## Introduction

Endosulfan (6, 7, 8, 9, 10,10-hexachloro-1, 5, 5a, 6, 9, 9a hexa hydro-6, 9-methano 2, 3, 4 benzo (e) dioxathiepen-3oxide) is an important chlorinated hydrocarbon pesticide chemically similar to aldrin, chlordane, and heptachlor, used against insect pest for a number of diverse applications [1] and is a neurotoxin which blocks the inhibitory receptors of the central nervous system, disrupts the ionic channels, and destroys the integrity of the nerve cells [2]. Poultry farming in India is an internal part of the agricultural industry [3,4]. Endosulfan and related insecticides have been used for the control of termites in chicken houses [5]. Many parts of the world have fallen prey to this pesticide that has affected a lot of humans, animals, and the environment. The Supreme Court of India had passed an interim order for the victims reported to be affected in Kasaragod (Kerala), and banned the production, distribution, and use of endosulfan. Thus, the present topic is important to decision making on further usage of endosulfan.

The continuous use of pesticides even in normal recommended doses may cause deleterious effect on the physiological functions [5-8] that may range from lower immunity of flock [9,10] to decrease production performances [11]. The lower immune competence in animals and birds due to environmental pollutant may lead to increase susceptibility [12,13], occurrence of reinfection, epidemics of disease [5,14,15], and vaccine failures [16] causing serious economic losses and thereby hampering the purpose of raising animals and birds. Hematological, biochemical, and pathological effect of chronic exposure to insecticides were also evaluated in indigenous chickens [17-20]. These studies in layer birds of India have not been reported intensively.

The present study was undertaken with the aim to study the effects of endosulfan on immunity in layers, and subsequently, its effect on immune status and teratogenic exhibits in chicks produced from these chlorinated hydrocarbon fed birds.

## Materials and Methods

### Ethical approval

The experiments were performed after obtaining permission from the Animal Ethics Committee. The regulations addressing animal use were followed and proper care and attention were given to the birds used in these experiments. Moribund birds and the birds at the end of the experiments were humanely sacrificed by cervical dislocation.

### Experimental design

A total of 20 White Leghorn layer birds of around 1 year age were selected for the present study at Poultry Research Centre, GB Pant University of Agriculture and Technology, Pantnagar. All the birds were maintained under standard and uniform conditions of feeding, management, and disease control under deep litter system of housing. The selected birds were vaccinated with Ranikhet vaccine F_1_ strain through intraocular route for both primary and booster dose. The chicks hatched out of endosulfan fed layer birds were also assessed for immunocompetence tests.

The endosulfan (35% E.C.) from Gujarat Pesticides Pvt., Ltd., Kalol, Gujarat, was procured from local market and mixed in drinking water. A solution was prepared for endosulfan by taking 10 ml original solution and 90 ml of water was added to it. From this solution, 9 ml was daily mixed in drinking water of birds (treatment) to give them desired concentration, i.e., 30 ppm per bird (no observable effect level dose as per long-term toxicity trials). Control birds were given normal water. The experiment was divided into two parts:

#### Effect of endosulfan in layers

In the first part of the experiment, 20 birds were divided at random into two groups, *viz*., control (C) and endosulfan-treated group (T_1_), each having 10 birds comprising of 8 hens and 2 cocks. Immunological and biochemical parameters in these layers were studied after collection of blood from these birds from wing vein at 15-day interval up to 90 days. The first moiety of blood was taken in heparinized vials (10-12 IU/ml) to study the biochemical parameters (serum total protein, albumin, globulin, and gamma globulin). The second moiety of blood was collected in sterilized vials for the collection of serum and further carrying out immunological studies and antibody titer through enzyme-linked immunosorbent assay (ELISA) using Ranikhet disease virus antigen [21]. The following immunological studies were carried out:

- Lymphocyte blastogenesis assay using Con-A and lipopolysaccharide (LPS) as mitogen [21]
- Total leukocytes count (TLC), absolute lymphocyte count (ALC), and absolute heterophil count (AHC) [22]
- Oxidative burst assay for macrophage activity [23].
- The biochemical parameters studied using serum was:
- Antibody titer through ELISA using Ranikhet disease virus antigen
- Biochemical test for measurement of total protein, albumin, globulin, and gamma globulin.

#### Effects of endosulfan in chicks hatched from endosulfan fed birds

The second part of the experiment included the study of immune competence status of chicks obtained from endosulfan fed birds. Thus, the chicks from each group were divided into three categories, i.e., chicks coming from birds after 1 month of endosulfan treatment, 2 months of endosulfan treatment, and the chicks coming from birds after 3 months of endosulfan treatment. Thus, all the chicks of each category obtain from both the group were maintained for 1 month. At 1 month of age, blood samples from all the chicks were collected directly from the heart into two parts: One portion was taken in heparinized vials to assess the hematological and immunological parameters, and another was taken in sterilized vials for serum collection. The hematological/immunological and biochemical parameters were studied in chicks to assess the teratogenic effect of endosulfan which were also studied in their parents. None of the chicks were treated with endosulfan.

### Statistical analysis

All data were expressed as mean±standard error. The statistical significance of the mean differences between control and treated groups was analyzed by Student’s t-test [24]. Statistical calculations were performed with the SPSS 13 computer program (SPSS Inc., Chicago, Illinois, USA). The value of p<0.05 was taken as the cutoff value to consider statistically significant differences.

## Results and Discussion

The endosulfan pesticides were found to have a depressive effect on total leukocytes and absolute lymphocyte counts after feeding for 90 days. These cells were found to be decreased up to 20% as compared to control (Table-1). Leukopenia may be due to cytotoxic effects of endosulfan, since the lymphocytes are the main cells to play the key role in defense mechanism, a reduction in the number of absolute lymphocytes as observed in the present study is an indication of immunosuppression. Earlier studies with lindane, quinalphos, carbaryl, fenvalerate, butachlor, and isoproturon revealed leukopenia and lymphopenia [25-27].

**Table-1:** Effect of endosulfan (mean±SE) on various leukocytic counts (10^3^/µl^−1^) in layers.

Days	TLC		ALC		AHC	
		
	C	T_1_	C	T_1_	C	T_1_
0	24.53±0.55	24.53±0.55	15.43±0.45	15.45±0.45	7.84±0.17	7.84±0.17
15	26.10±0.27	24.08±0.25*	16.18±0.12	15.00±0.16	7.83±0.21	7.86±0.32
30	27.30±0.22	24.76±0.23*	18.55±0.89	15.92±0.12*	7.53±0.52	7.59±0.59
45	28.99±0.19	24.86±0.17*	20.20±0.22	16.07±0.19*	7.53±0.15	6.95±0.20
60	30.29±0.20	26.74±0.24*	22.49±0.32	18.00±0.23*	6.66±0.46	7.31±0.22
75	31.04±0.13	29.03±0.30*	23.59±0.13	19.54±0.81	5.83±0.11	8.22±0.32*
90	31.93±0.31	28.87±0.25*	25.01±0.28	19.91±0.16*	6.82±1.14	7.98±0.12

* Significant difference from control within column (p≤0.05). SE=Standard error, TLC=Total leukocyte counts, ALC=Absolute leukocyte counts, AHC=Absolute heterophil count

Birds immunized with Ranikhet disease vaccine and exposed to endosulfan showed a marked decrease in delta optical density (OD) after stimulation with mitogen Con-A, which indicates lowered cellular immune response. Decrease in the lymphocyte proliferation by mitogen Con-A (14.23% Table-2) is an indication of suppression of T-cell blastogenesis, which is essential for mounting of both the cell-mediated and humoral immune response. Earlier, workers have also reported [28] reduced lymphocyte blastogenesis response in minks and ferrets exposed to hexachlorobenzene. Depression in cell-mediated immune response in pesticide fed birds, as observed in this study, has also been reported in broilers as measured by lymphocytes migration inhibition test [29] and lymphocyte stimulation test (LST) using 3-(4,5-dimethylthiazol-2-yl)-2,5-diphenyltetrazolium bromide dye method [25,27,30].

**Table-2:** Effect of endosulfan (mean±SE) on Cell-mediated and humoral immune response in layers.

Days	Cell-mediated immune response				Humoral immune response			
	
	T-lymphocyte blastogenesis (delta OD)		OBA (NO-_2_−production µM/ml)		Humoral immunity (ELISA values)		B-lymphocyte blastogenesis (delta OD±SE)	
			
	C	T_1_	C	T_1_	C	T_1_	C	T_1_
0	0.20±0.025	0.20±0.03	190.40±22.78	190.40±22.78	1.52±0.26	1.52±0.26	0.16±0.090	0.16±0.09
15	0.19±0.015	0.20±0.03	215.20±30.70	167.03±25.41	2.55±0.06	2.52±0.02	0.20±0.08	0.18±0.01
30	0.21±0.05	0.20±0.05	346.28±33.12	288.53±32.99	2.45±0.04	2.43±0.06	0.25±0.02	0.19±0.03
45	0.26±0.03	0.23±0.10	181.96±26.72	129.27±18.42	2.40±0.09	2.25±0.09	0.32±0.06	0.23±0.05*
60	0.28±0.06	0.24±0.02*	311.31±36.98	236.57±34.55*	2.30±0.07	2.01±0.07*	0.33±0.05	0.24±0.08*
75	0.29±0.04	0.25±0.02*	132.36±22.53	43.74±7.14*	2.29±0.08	1.95±0.08*	0.34±0.09	0.24±0.06*
90	0.30±0.059	0.26±0.03*	316.04±34.61	247.00±30.83*	2.25±0.05	1.90±0.05*	0.35±0.02	0.25±0.04*

* Significant difference from control within column (p≤0.05). SE=Standard error, OBA=Oxidative burst assay, OD=Optical density

Nitrous oxide (NO) is a gaseous, free radical molecule, which is catalytically generated by the cellular NO synthetase from L-arginine to L-citrulline. Besides being an essential neurotransmitter, NO or its derivatives have inhibitory effects on a variety of infections [31]. In the present investigation, the production of nitric oxide in avian macrophage culture was found to be reduced as a result of endosulfan treatment. The reduction of NO varied in a dose-dependent manner. When macrophages are activated with bacterial lipopolysaccharides, the expression of high levels of nitric oxide synthetase occurs that oxidized L-arginine to L-citrulline and NO gas [32]. NO has potent antimicrobial activity and even it can combine with superoxide anion to yield more potent antimicrobial activity against, bacteria, fungus, and other pathogens [33]. The reduction in NO production as revealed in the present study, i.e., 21.84% (Table-2) in endosulfan fed birds lead to a state of decreased phagocytosis and immunosuppression.

Significant depression in Ranikhet disease vaccine-induced humoral immune response as measured by ELISA has been observed in the present study in birds exposed in comparison to control. The decrease in ELISA values (15.54% Table-2) was observed in birds fed with endosulfan in comparison to controls. The immunosuppressive action of pesticides is due to their detrimental action on lymphoid organs. The decreased number of lymphocytes along with decreased functional capacity has been reported in patients following pesticide intoxication [34]. The decreased activity of T-lymphocytes might affect T-cell-dependent humoral immune response also.

The reduction in antibody titer was further confirmed by decreased serum gamma globulins and decreased activity of B-lymphocyte blastogenesis by 27.82% (Table-2) in endosulfan fed birds. The gamma globulins are directly related to the antibody titer measured by ELISA, which were found to be significantly decreased in the endosulfan-treated group. The 35.71% decrease in serum gamma globulin (Table-3) is an indication of lowered immunity, whereas ELISA detected the reduction in specific antibody titer to Ranikhet disease vaccine. A decrease in serum globulin and immuno-globulin levels has been reported in rabbit and mice due to Aroclor and DDT [35], in rats due to lindane [36] in birds due to carbaryl, malathion [29], cypermethrin [30], gamma benzene hexachloride and quinalphos [25], butachlor and isoproturon [27]. Prenatal exposure of carbofuran or diazinon results into alterations in immunoglobulins levels in offsprings of mice [37]. The decrease in serum gamma globulin contents might be due to the effect of pesticides on peripheral blood lymphocytes, which were found to be decreased in the present study. Since the gamma globulins are synthesized by the lymphocytes and pesticides being lymphocytotoxic, the quantitative reduction was evident because of their effect on peripheral blood lymphocytes [5]. Reduction in serum gamma globulin concentration might also be due to the immunosuppressive effect of insecticides on antibody production or due to functional impairment of B-lymphocytes.

**Table-3:** Effect of endosulfan (mean±SE) on serum proteins in layers.

Days	Serum albumin (mg/dl)		Serum globulin (mg/dl)		Gamma globulin (g/100 ml)		Serum total proteins (g/100 ml)	
			
	C	T_1_	C	T_1_	C	T_1_	C	T_1_
0	2.50±0.31	2.50±0.31	1.87±0.32	1.87±0.32	1.30±0.32	1.30±0.32	4.37±0.18	4.37±0.18
15	2.27±0.34	2.01±0.42	2.23±0.19	1.89±0.37*	1.56±0.17	1.28±0.19	4.50±0.18	3.90±0.18*
30	2.35±0.17	2.00±0.19	2.65±0.20	2.00±0.22*	1.85±0.37	1.36±0.40*	5.00±0.20	4.00±0.46*
45	2.40±0.32	2.12±0.18*	2.90±0.27	2.08±0.12*	2.03±0.40	1.41±0.39*	5.30±0.13	4.20±0.37*
60	2.70±0.32	2.27±0.29*	2.30±0.39	1.92±0.25*	1.61±0.36	1.30±0.22	6.00±0.32	4.19±0.32*
75	2.90±0.09	2.35±0.18*	3.60±0.22	2.65±0.19	2.52±0.19	1.59±0.17*	6.50±0.36	5.00±0.40*
90	3.00±0.19	2.50±0.38*	4.00±0.17	2.82±0.39	2.80±0.22	1.80±0.31*	7.00±0.50	5.32±0.41*

* Significant difference from control within column (p≤0.05). SE=Standard error

The total serum proteins, serum albumin, and serum globulin values were significantly less in endosulfan fed birds as compared to control birds (Table-3). Similarly, decreased serum proteins were also observed in malathion fed birds [5,29] and goats fed carbaryl [38]. A decrease in serum proteins was reflected due to decrease in albumin and gamma and beta globulin values in rabbits administered dichlorodiphenyltrichloroethane [39] and in buffalo calves sprayed with deltamethrin [40].

In the present study, the results on effect of endosulfan on chicks hatched from endosulfan fed birds revealed the non-significant difference between groups for all the hematological, biochemical, and immunological parameters studied (Tables-4 and 5). The values for all the hematological parameters TLC, ALC, and AHC were approximately similar in control and treated groups of chicks (Table-5). Similarly, the values for biochemical parameters serum total proteins, albumin, globulin, and gamma globulin were also approximately same for control and endosulfan-treated groups. The values for immunological status (LST and ELISA) were also approximately similar in control and endosulfan-treated group of chicks.

**Table-4:** Teratogenic effect of endosulfan (mean±SE) on Cell-mediated and humoral immune response in chicks.

Months	Cell-mediated immune response		Humoral immune response			
	
	T-lymphocyte blastogenesis (delta OD)		B-lymphocyte blastogenesis (delta OD)		Humoral immunity (ELISA values)	
		
	C	T_1_	C	T_1_	C	T_1_
1^st^	0.20±0.27	0.25±0.20	0.150±0.32	0.130±0.27	0.74±0.14	0.65±0.63
2^nd^	0.29±0.32	0.20±0.13	0.350±0.17	0.380±0.24	0.56±0.41	0.53±0.10
3^rd^	0.27±0.18	0.23±0.19	0.094±0.26	0.094±0.70	0.63±0.10	0.92±0.28

SE=Standard error, OD=Optical density, ELISA=Enzyme-Linked immunosorbent assay

**Table-5:** Teratogenic effect of endosulfan (mean±SE) on various leukocytic counts (10^3^/µl) in chicks

Months	TLC		ALC		AHC	
		
	C	T_1_	C	T_1_	C	T_1_
1^st^	22.39±0.34	21.27±0.50	13.80±0.27	12.97±0.29	7.01±0.62	6.59±0.27
2^nd^	21.32±0.52	20.67±0.31	13.37±0.17	12.48±0.60	6.77±0.53	6.81±0.18
3^rd^	21.40±0.61	20.94±0.35	13.04±0.25	12.91±0.20	6.63±0.31	6.56±0.15

SE=Standard error, TLC=Total leukocyte counts, ALC=Absolute leukocyte counts, AHC=Absolute heterophil count

## Conclusion

It can be concluded from the present study that there were leukopenia and lymphopenia in endosulfan-treated birds with decrease number of heterophils. Similarly, there was reduction in delta OD of the lymphocytic cultures stimulated by Con-A and LPS mitogens indicating a decrease in cellular and humoral immune response in endosulfan fed birds. Total serum protein, serum albumin, serum globulin, and serum gamma globulin were significantly decreased in endosulfan fed birds. Antibody titer against Ranikhet disease vaccine as measured by ELISA decreased significantly in endosulfan fed birds as compared to control suggesting cautious usage of feeds, in which endosulfan is present in the residual form. Since various crops, which are used in poultry feeds, are protected by pesticides (endosulfan) during their cultivation practices, contamination through this chain is easily possible and birds can get prey to it.

## Authors’ Contributions

PPS, AK, and RSC designed the work. PPS conducted the study. PPS and PKP helped for statistical analysis. PPS and PKP prepared the manuscript. PKP revised the manuscript for communication to the journal. All authors read and approved the final manuscript.
